# Digital Health Center App for Community and National Malaria Surveillance in Cambodia: Implementation Case Study

**DOI:** 10.2196/85881

**Published:** 2026-06-12

**Authors:** Pengby Ngor, Huy Rekol, Eng Thyda, Ry Rith, Sok Kimleng, Yoem Rattana, Ou Vunsokserey, Hem Vanna, Chang Pumeoung, Pascal Ringwald, Richard J Maude, Lisa White, Siv Sovannaroth

**Affiliations:** 1 National Center for Parasitology, Entomology and Malaria Control, No. 477, Betong (St.), corner of St. 92, Trapeang Svay Village, Phnom Penh, 12101, Cambodia, 855 889989899; 2 Mahidol Oxford Tropical Medicine Research Unit, Bangkok, Thailand; 3 The Open University, Milton Keynes, United Kingdom; 4 WHO Mekong Malaria Elimination Programme, Phnom Penh, Cambodia; 5 Department of Medicine, Centre for Tropical Medicine and Global Health, University of Oxford, Oxford, United Kingdom; 6 Model Health Ltd, Brixham, United Kingdom

**Keywords:** malaria, Cambodia, surveillance, elimination, information systems

## Abstract

**Background:**

By 2015, the emergence and dissemination of multidrug-resistant *Plasmodium falciparum* in the Greater Mekong Subregion threatened regional and global malaria control efforts. In response, Greater Mekong Subregion countries committed to malaria elimination by 2030, with strengthened surveillance as a strategic pillar. In 2017, Cambodia introduced an elimination-oriented digital Malaria Information System (MIS). Its health center app enables real-time, geo-located, case-based malaria reporting across primary health centers, and is fully integrated with the MIS.

**Objective:**

This study aimed to evaluate the real-world national implementation of Cambodia’s Android-based health center app, considering coverage, fidelity, timeliness, and data use, and their effects on malaria surveillance performance, case management, programmatic response, and public health outcomes.

**Methods:**

System performance and public health use were assessed using system-generated metadata, national surveillance data, and user surveys. Operational indicators included technical performance, data completeness, and reporting timeliness, alongside surveillance outcomes such as case notification, classification, reactive case detection, and foci investigation. Nationwide user experience was measured via a survey of 761 health centers across 21 provinces, with in-depth structured surveys at 9 health centers in 3 provinces. Descriptive analyses evaluated system functionality, contribution to malaria surveillance and response, and usability among frontline health workers.

**Results:**

The health center app demonstrated strong technical performance, with rapid loading and resilient data transmission under low-bandwidth conditions, supporting reliable reporting in resource-constrained settings. Integrated real-time dashboards provided analytics for case management, surveillance monitoring, risk stratification, and targeted public health interventions. Data completeness remained high (99%, 89/90 fields in 2024), demonstrating consistent routine use even as case incidence declined. Between January 1, 2025, and July 31, 2025, 69 malaria cases were reported nationally (23 locally acquired, 7 domestically imported, and 39 internationally imported). Of these, 95.7% (66/69) were notified and classified within 1 day. Reactive case detection was completed within 3 days for all 21 eligible cases, and 16 of 19 eligible foci received a response within 7 days, indicating strong operational responsiveness. User surveys showed 96.3% (733/761) of health centers were satisfied or very satisfied, 90.1% (686/761) reported rare or no technical issues, and 91.7% (698/761) found the app easy to navigate. Operational challenges included limited internet connectivity, transport to remote areas, and electricity interruptions. In-depth surveys confirmed high uptake, confidence in reporting, and routine use of surveillance data, although gaps in local analytical capacity were identified.

**Conclusions:**

Developed and managed locally to enhance sustainability, the MIS drove significant reductions in malaria case incidence, with the health center app contributing timely, complete, structured reporting at the point of care. Public health responses were facilitated by real-time analysis, targeted interventions, and decentralized decision-making. User engagement was sustained as malaria cases declined, and further enhancements are planned to ensure seamless transition to postelimination surveillance, reducing the risk of malaria reestablishment in Cambodia.

## Introduction

Malaria is a life-threatening parasitic disease caused by *Plasmodium spp*., transmitted to humans through the bites of infected *Anopheles* mosquitoes. Malaria continues to pose a major global health challenge, causing significant morbidity and mortality [1].

The Greater Mekong Subregion (GMS) has been a focus for the development of parasite resistance to antimalarial drugs. In the late 1950s, chloroquine-resistant *Plasmodium falciparum* emerged in the GMS, and over the following 2 decades disseminated worldwide [2]. This led to extensive treatment failures and significantly undermined malaria control programs globally [3,4]. Similarly, the GMS was the source of pyrimethamine-resistant parasites which quickly spread to Africa [5]. The introduction of artemisinin-based combination therapy (ACT) in 2001 marked a turning point in malaria control, restoring treatment efficacy and becoming the cornerstone of global treatment strategies. Consequently, reports in 2008 of the emergence of *P. falciparum* artemisinin partial resistance on the Thai-Cambodia border generated considerable concern. The subsequent evolution of multiresistant parasites and their rapid proliferation across the GMS complicated efforts to treat and contain the disease [6-8]. There was a considerable risk that these multiresistant parasites would spread globally, causing malaria to become untreatable [9]. This led to urgent and coordinated regional efforts to contain malaria [10], which by 2015 had evolved into a commitment to attain malaria elimination in the entire GMS by 2030 [11].

Malaria elimination is the interruption of local transmission in a defined geographical area [12]. It is an ambitious objective that is complex to achieve. Elimination is pursued in phases [11,12]. In the transmission-reduction phase, broad interventions are applied to reduce disease burden, such as widespread vector control, including the distribution of insecticide bed nets, and strengthening malaria services to deliver effective medicines. Once case incidence is low (typically below 1 case/1000 people/year), the elimination phase begins, and surveillance becomes the primary intervention [11,12]. The aim is to systematically drain the human transmission reservoir by finding and eliminating all parasites and interrupting transmission. To achieve this, a strong and responsive surveillance system is needed, capable of promptly detecting and treating every case, determining the origin of each case, triggering a timely and appropriate public health response, and detecting any changes in therapeutic drug efficacy or transmission patterns. This is generally achieved via one or more of three main surveillance strategies:

1. The 1-3-7 strategy: supporting malaria elimination in China and elsewhere, each case is reported within 1 day, investigated within 3 days, and followed by a targeted response within 7 days to prevent onward transmission [13-15].
2. Integrated drug efficacy surveillance (iDES): an early warning of drug resistance to ensure effective treatment; follow-up is extended to Day 42 for *P. falciparum* and Day 90 for *P. vivax* cases to identify treatment failures; blood spot samples are also collected for molecular analysis of known drug resistance markers [16-19].
3. Stratification of malaria risk: detailed epidemiological and ecological data at a high level of granularity are used for targeting to ensure effective deployment of essential resources.

Although these strategies can be implemented independently, their integration strengthens malaria surveillance and response systems by linking timely case detection, investigation, targeted intervention, and drug efficacy monitoring. This ensures good patient outcomes, reduces transmission potential, and maximizes the public health benefit. A coordinated approach also reduces duplication of effort, improves the efficiency of resource use, and enhances the sustainability of malaria elimination programs.

**Figure 1.**
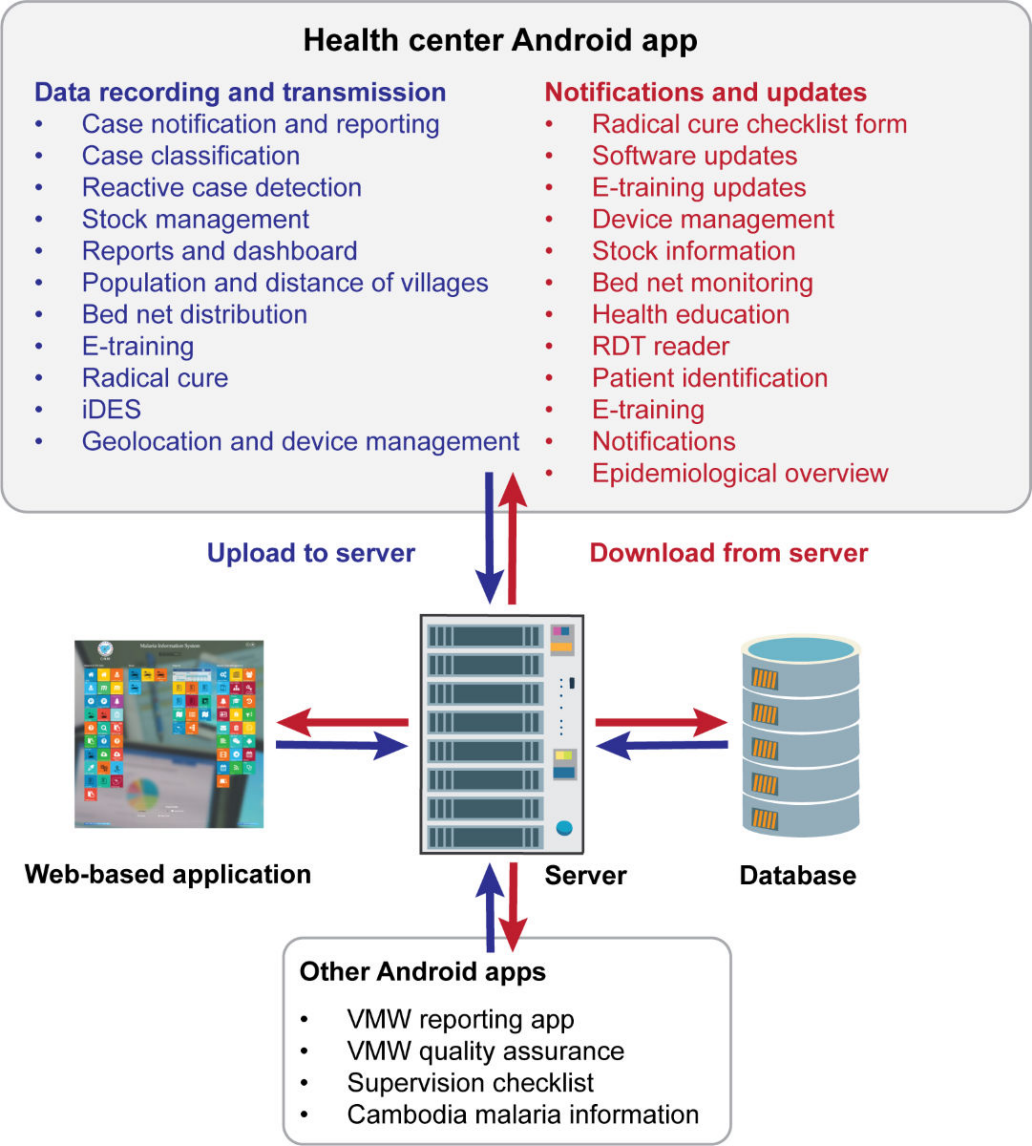
Overview of health center app within the Cambodian malaria surveillance system. iDES: integrated drug efficacy surveillance; RDT: rapid diagnostic test; VMW: village malaria worker.

In Cambodia in 2008, malaria surveillance was severely limited and ineffective malaria treatments were commonly prescribed, deepening the public health impact and fueling transmission. Initial efforts to contain artemisinin resistance included banning artemisinin monotherapies in favor of more effective ACTs, phasing out the private sector, deploying insecticide-treated bed nets, facilitating information sharing across borders, and introducing malaria surveillance using a Microsoft Access Database. As the regional response pivoted to target malaria elimination, Cambodia implemented a National Strategic Plan for Malaria Elimination (2011‐2025). This included substantial investments in surveillance infrastructure by the Cambodian Ministry of Health, led by the National Center for Parasitology, Entomology and Malaria Control (CNM) [20,21]. These efforts expanded surveillance activities via the Malaria Information System (MIS), which was migrated to a web-based platform in 2015 [22,23]. However, data were still collected on paper forms, with aggregation at the operational district level and monthly reporting. This process was slow, data were often incomplete, and outputs lacked the required granularity to support elimination efforts within an increasingly heterogeneous and fragmented transmission landscape [22-24]. To overcome these limitations, a fully digital system was launched in 2017, combining web-based analytics on the MIS, with 2 mobile apps enabling digital real-time reporting from health workers in the field, one for village malaria workers (VMWs) and another for staff at health centers (Figure 1) [24,25]. The MIS thereby supports the 1-3-7 strategy, iDES, and malaria risk stratification within one fully integrated platform extending throughout the public health system and the community.

Cambodia’s national malaria surveillance system has been widely recognized internationally as a model for digital transformation in elimination settings [26-31]. Efficient surveillance has supported an extraordinary decline in malaria cases from over 66,000 in 2018 to 355 in 2024, with no deaths from malaria recorded over this period [1,25,32]. Given that malaria elimination remains a priority for the World Health Organization and the Global Fund to Fight AIDS, Tuberculosis and Malaria, documentation of a fully implemented, nationally integrated digital surveillance platform has substantial global relevance. The broader surveillance architecture and its evolution have been described in recent publications [24,25]. This study extends that work by documenting the integration of the health center-level digital surveillance application into the country’s malaria surveillance architecture. Operationalization of the system at the primary health care level is essential, as real-time case reporting, treatment verification, and response coordination directly influence elimination outcomes. This implementation case study focuses on a snapshot of recent data to highlight both the strengths and limitations of the health center app as Cambodia strives to complete the “last mile” to malaria elimination. The architecture and implementation principles are directly transferable to other malaria-endemic countries transitioning from control to elimination, focusing on practical insights for national-level digital health surveillance planning and sustainability in preelimination settings.

## Methods

### Ethical Considerations

This work was conducted as part of CNM’s ongoing efforts to strengthen routine surveillance, drawing on existing program data and surveys with VMWs about their professional use of a government-supported malaria reporting system. All respondents were public health personnel acting in their official roles, and participation in the surveys was voluntary and anonymous. No information that could identify individual patients or reveal personal health details was collected or assessed. Under national guidelines, the activities did not require formal ethics committee review or written informed consent. All procedures complied with relevant institutional, national, and international standards for data protection. No formal ethics committee approval was sought for this work because the project constituted a service implementation and evaluation activity using routinely collected, fully anonymized patient data, with no direct patient contact or intervention. Staff participants contributed only in their professional capacities as employees of the digital health service. Guidance from the University of Cambridge Research Ethics framework states that ethical review is not normally required for the secondary use of robustly anonymized data where individuals are not identifiable to the research team. Relevant guidance is available from the University of Cambridge Research Ethics Handbook [33] and associated ethics review guidance: University of Cambridge Ethics Review Flowchart [34]. Cambodian national health research ethics oversight information is available from the Cambodian National Ethics Committee for Health Research [35].

### Malaria Surveillance in Cambodian Health Centers

Health centers serve as the primary formal health care contact for rural populations, offering essential diagnosis, treatment, and referral services for common illnesses. Each center covers a defined catchment area, typically including 8000‐12,000 persons, and operates under Ministry of Health guidelines. For malaria, health center staff use rapid diagnostic tests (RDTs), prescribe ACTs, and record clinical and epidemiological details of each case. Health centers also provide support for VMWs, compile routine reports, and conduct follow-up investigations to direct local malaria responses.

Since 2017, health centers have conducted real-time case-based reporting via a dedicated digital application (Figure 1). Health center participation is mandatory in endemic districts, with malaria designated as a notifiable disease, supporting broad coverage and adherence to reporting requirements. Consequently, health center data form a core component of Cambodia’s malaria surveillance infrastructure, contributing to individual case management, targeted local responses, and national-level monitoring and elimination planning.

### App Development

The health center mobile app was developed in-house by CNM, with support from the Global Fund, to strengthen malaria elimination activities. Its primary aim is to enable timely, accurate, standardized malaria case reporting from public health centers, aligning with national strategy [20,21].

Built as a native Android app using Java, the app was designed for frontline health staff, considering feasibility, usability in low-connectivity settings, and compatibility with Ministry of Health workflows. Supporting Android versions 5.0+, this platform was selected over iOS and web-based alternatives due to its affordability, flexibility, offline functionality, and compatibility with the low-cost, older smartphones and tablets available within the public health system.

Recognizing the limited network access in rural areas, the app features offline-first functionality with local SQLite data storage, synchronizing automatically to the central MIS (Microsoft SQL Server) when internet becomes available (Figure 1). This integration supports near real-time data consolidation with other sources (eg, VMW app). The MIS also automatically shares data with the National Health Information System and the Malaria Reference Laboratory, with population information maintained through the Ministry of Planning Commune Database.

**Figure 2.**
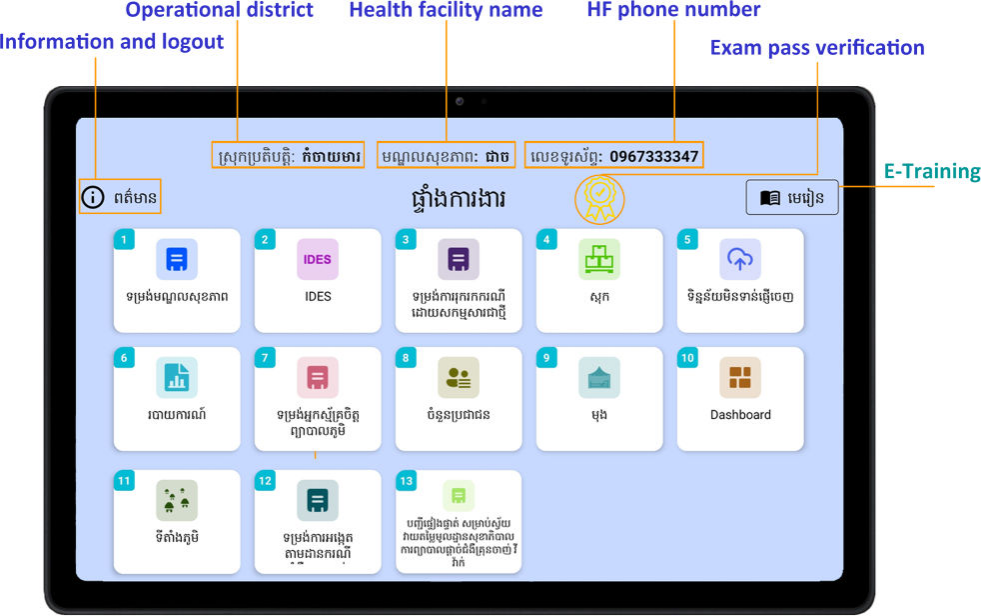
Health center app graphical user interface home screen. Top right: E-training – online resources and training materials and courses. (1) Patient reporting; (2) Integrated drug efficacy surveillance (iDES); (3) Reactive case detection; (4) Stock management; (5) Offline mode – management of data transfer offline and online; (6) Reporting; (7) Village malaria workers; (8) Population; (9) Bed-net status; (10) Dashboard; (11) Village location; (12) Case classification; (13) *P. vivax* radical cure. The figure was derived with permission from CNM’s online malaria information system available via their website [36].

A user-centered design approach guided development. The interface uses icons to enhance usability and organizes functions into distinct modules (Figure 2). The system brings together a comprehensive suite of malaria surveillance and management functions, beginning with patient reporting, which captures essential epidemiological case data at the point of care. Additionally, the system supports detailed case classification, distinguishing local from imported cases and further categorizing indigenous, induced, recrudescent, and relapsing infections. It supports timely reactive case detection to actively identify additional infections around confirmed cases that might otherwise be missed and enables effective management of radical cure for *P. vivax* malaria, including tracking treatment eligibility and implementation across health facilities. Demographic insights are strengthened through population data that help determine accurate incidence rates, complemented by bed-net status features that record distribution dates, coverage, and expiry alerts. An iDES module is included to provide early warning signals of emerging drug resistance through extended follow-up of *P. falciparum* and *P. vivax* cases. Operational efficiency is supported by stock management tools that monitor commodity levels, expiry dates, and re-ordering needs, while an offline mode ensures continuous data capture in low-connectivity settings. The platform also offers robust reporting capabilities through automated and customizable analytics and facilitates oversight of VMWs by tracking their locations, training, reporting performance, and supervision. A centralized dashboard synthesizes key indicators to present a clear picture of the local malaria situation with precise village location mapping assigned using standardized GPS-based codes.

Prototypes underwent scenario-based testing at CNM with iterative refinement involving health staff, CNM surveillance officers, and district managers, ensuring usability and relevance. Updates continue based on input from subnational teams and evolving program needs. A dedicated 7-member CNM team manages maintenance, updates, training, and technical support. A custom device management module enables remote software updates, monitors device use, and helps resolve hardware issues. Health centers can also report lost or damaged devices directly to CNM.

### Data Processing

To facilitate adoption and reduce training needs, the app replicates the national paper-based malaria reporting forms used at health centers, aligning with national surveillance standards. Structured digital forms feature drop-down menus, prepopulated fields, and built-in logic to minimize errors and ensure consistency. This familiar design supports harmonized data collection, standardized case classification, and seamless analytics for trend monitoring and outbreak detection. Automated data quality checks verify completeness and internal consistency. In-app alerts notify users of missing fields or late submissions, while recent entries can be reviewed for prompt correction, reinforcing accountability. Entries are time-stamped and geotagged using built-in GPS functionality, enabling fine-scale spatial mapping, real-time risk stratification, and targeted response activities.

Once uploaded, case data feeds into the MIS, which applies validation checks and flags cases requiring follow-up. The MIS generates visual outputs, including time-series graphs, incidence maps, and summary tables, accessible to national and subnational teams via a secure online interface to track surveillance quality, treatment timelines, and emerging hotspots. To enhance performance in low-bandwidth areas, the MIS uses pregenerated dashboards and analytics that refresh hourly. Visuals are cached and compressed for fast access on both desktop and mobile devices, even with limited connectivity.

### Data Security

Security and data protection were integrated from the design stage. Data transmitted between the app and MIS server is encrypted via HTTPS, with user access controlled through unique logins authenticated against CNM’s central system. To safeguard patient confidentiality, personally identifiable information is minimized and retained only at the local level for follow-up care. Each case is assigned a unique identifier, and data access is role-based. CNM uses basic pseudonymization for national reporting, aligning with international standards for data minimization and secure storage.

### Implementation

**Figure 3.**
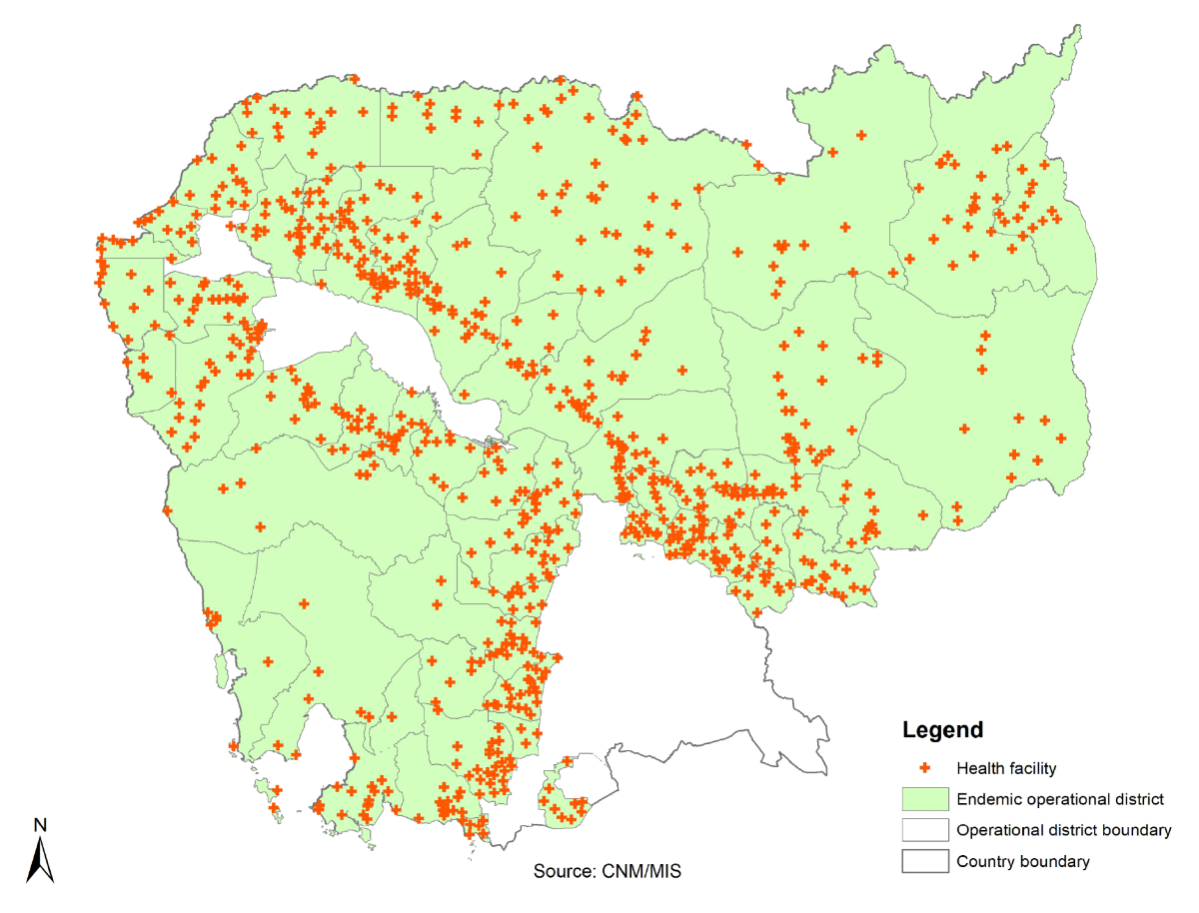
Distribution of health centers using the mobile app in 2024. The figure was derived with permission from CNM’s online malaria information system available via their website [36].

In 2017, CNM procured 800 tablets for distribution to health facilities across 21 provinces, completing rollout by early 2018. Since 2021, the app has been running on general-use health center devices, improving sustainability. As of June 2025, there are 977 devices active across 871 health centers, recognizing that most health centers now have more than one device (Figure 3).

Initial deployment included in-person training, followed by field visits for reinforcement and troubleshooting. New staff are onboarded through a similar process, with training typically requiring 2 hours. Annual refresher sessions are held to maintain user skills. CNM also provides ongoing support via phone or Telegram, with on-site visits for unresolved issues.

To enhance continuous learning, the app includes an embedded e-training module offering videos, slides, checklists, and quizzes. This enables staff to troubleshoot issues independently and assess their knowledge. CNM can remotely update training materials and send reminders to ensure compliance. For example, during the COVID-19 pandemic, the e-training module allowed uninterrupted training while supporting infection control. In particular, advice could be updated on how to continue to deliver malaria services effectively during the pandemic and differential diagnosis of fever. Additionally, an in-app messaging feature enables health workers to submit questions directly to CNM, which also helps identify broader knowledge gaps.

### Monitoring and Evaluation

A comprehensive evaluation and monitoring framework assesses the performance, usability, and programmatic use of the health center app. This framework combines continuous system-generated monitoring, routine surveillance validation, user feedback mechanisms, and periodic in-depth assessments to ensure the system remains aligned with malaria elimination requirements and responsive to user needs.

Technical performance is monitored continuously using automated metadata collected through the app and the MIS. Key performance indicators include app launch time, data upload and download success rates, synchronization times under varying network conditions, and crash frequency. A third-party monitoring service (Firebase Crashlytics) generates real-time alerts enabling rapid identification and rectification of technical issues. Timing logs capture request–response durations, supporting evaluation of system resilience under low-bandwidth conditions. These real-time indicators allow the CNM to assess system reliability, detect emerging technical constraints, and prioritize software improvements.

Routine monitoring includes tracking the completeness, accuracy, and timeliness of case-based reporting. Data submitted through the app are automatically transferred to the MIS, where automatic validation checks assess internal consistency, missing data, and unusual entries. Unusual or inconsistent patterns are flagged and followed up with field visits. Health center participation rates, frequency of app use, and completeness of mandatory fields are also monitored. Evaluation of case form content includes verification of demographic, diagnostic, and treatment variables. Malaria elimination surveillance performance is assessed through real-time longitudinal monitoring of the 1-3-7 framework, iDES, and foci clearance. The app also supports risk stratification at the village level, a key programmatic tool.

Ongoing evaluation of the health center app’s usability and contextual performance is essential. Real-time feedback is routinely collected, and user surveys are conducted periodically to check alignment between the app functionality, user experiences, and programmatic objectives. More in-depth surveys are conducted to obtain fine-grained information on potential app improvements and training needs.

## Results

### Data Collection for Case Management

Data submitted through the health center app are automatically transmitted to the MIS, where they undergo routine validation and analysis. These data underpin surveillance outputs guiding malaria elimination across all health system levels. In 2024, 99% (871/882) of endemic-area health centers used the app, logging an average of 4 visits per month. Data quality was high, with 99% (89/90) of submitted records complete and accurate.

**Figure 4.**
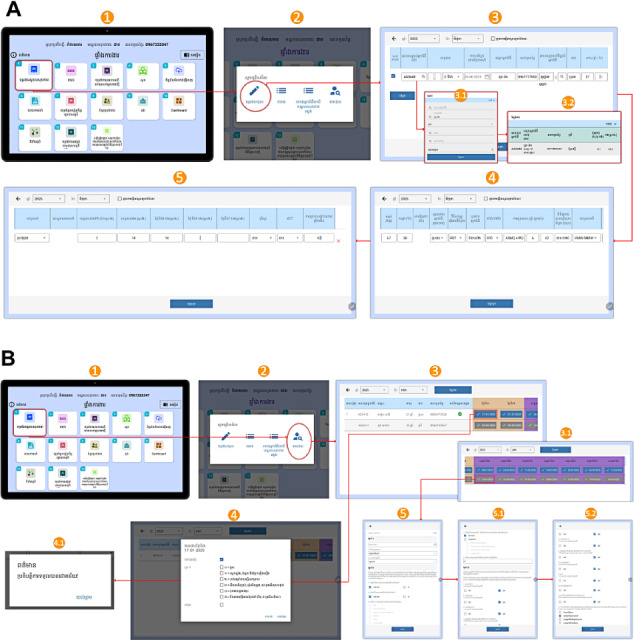
Examples of reporting workflow on the health center app. A: Case reporting workflow on the health center app. Starting from the health center app main menu health center form (1), the menu for data entry is selected (2), the month is selected and patient details are entered, including demographic and clinical characteristics, diagnostic test results, treatment and referral details (3); data can be linked to previous records using the patient code, name, sex, age, or contact details (3.1) and patient details can be viewed (3.2) with any additional patient details completed (4), and further details on treatment, referrals, and pregnancy, hemoglobin, and glucose-6-phosphate dehydrogenase test results as well as drug doses recorded (5). B: Follow-up schedule for *P. vivax* radical cure on the health center app. Starting from the health center app home menu health center form (1), the data entry menu is accessed, including *P. vivax* radical cure follow-up (2). Year and month are used to search for patient information (3) and follow-up is scheduled depending on treatment regimen (3.1). At follow-up, the date and time are noted and the patient asked about possible signs of hemolysis (paleness, yellow skin and eyes, shortness of breath, etc) (4). Treatment follow-up by the village malaria worker is detailed in section (5), providing information on adherence, recovery, and adverse events. The figure was derived with permission from CNM’s online malaria information system available via their website [36].

The malaria case form captures all essential data, including demographics, diagnostic methods and results, treatment regimens, and epidemiological risk factors such as travel, forest exposure, or occupation to inform accurate case classification (Figure 4A). Each patient has a unique code, confirmed with fingerprint identification, allowing linked interactions for follow-up and retreatment (Figure 4A). Accurate time and location information allows cases to be tracked in real-time and supports the deployment of rapid responses to prevent or contain outbreaks.

As malaria elimination has progressed in Cambodia, the epidemiological context has shifted from a high burden of *P. falciparum* malaria to most cases now caused by *P. vivax*. The treatment protocol for *P. vivax* is complex, requiring prior testing to determine glucose-6-phosphate dehydrogenase (G6PD) activity before treatment with an ACT for 3 days plus daily primaquine for 7 days for patients with normal or intermediate G6PD activity, or weekly primaquine for 8 weeks for patients with G6PD deficiency. Therefore, a specific workflow is incorporated to support efficient case management of *P. vivax* malaria cases, including follow-up and adherence (Figure 4B).

The app also provides individualized patient tracking, highlighting follow-up needs and adherence checks to support quality case management and programmatic objectives. Automatic reminders for follow-up interventions (phone calls or in-person visits) are generated, for example, to track patients enrolled in iDES or to support *P. vivax* radical cure adherence.

### Data Analytics for Malaria Elimination

To support immediate data use by frontline staff, the app includes an integrated dashboard showing key indicators by catchment area. Performance against the 1-3-7 framework is tracked with visualizations of cases disaggregated by *Plasmodium spp*., age, and sex using time-series graphs. Comparative summaries with peer facilities promote data use and performance monitoring. Automated flags identify cases for investigation. For example, delayed treatment suggesting access issues, retreatment possibly due to drug resistance, or cases in elimination zones triggering outbreak responses.

In addition to case data, the dashboard tracks preventive measures. Bed-net distribution status, including maps and expiry dates, supports redeployment planning. Also, procurement is guided by commodity stock level monitoring, with analytics identifying overstocked, adequate, or depleted sites. By delivering timely, accessible, and actionable insights, the dashboard transforms surveillance into a 2-way process, embedding data-driven decision-making into routine care and elimination activities.

### System Performance

In terms of technical operation, the system is robust, with key technical performance indicators shown in Table 1. The app opens rapidly, typically within 3 seconds. This ensures that the app is available at the time of patient consultation and is quickly accessible given the workload and time pressure that health staff often face. Data uploads and downloads are resilient, even on older 2G and 3G networks. Success rates for uploads (95%) and downloads (98%) are high, providing reassurance that the design of the system has been optimized for a low-bandwidth setting.

**Table 1.** Key indicators of health enter app technical operation.

Indicators	Outcomes
Time between app data submission and updated dashboard analytics availability, median (IQR)^a^	3 (3‐20)
Time taken for the app to launch and become usable after being opened, median (IQR)^a^	3 (3‐20)
Data uploads and downloads that were successful without errors or retries (%)	
Upload success rate	95
Download success rate	98
Proportion of attempts that required retries due to network failure, timeouts, or other issues (retry rate per 1000 operations)	50
Interactions that occurred in offline mode and later synchronized successfully (%)	10
Number of crashes (per 1000 active user-days)	20‐30
Time taken for synchronization segmented by network, median (IQR)^a^	
2G	13 (13‐30)
3G	10 (7‐30)
4G	3 (4‐20)
Wi-Fi	3 (4‐20)

a Measured in seconds.

### Surveillance Performance

**Figure 5.**
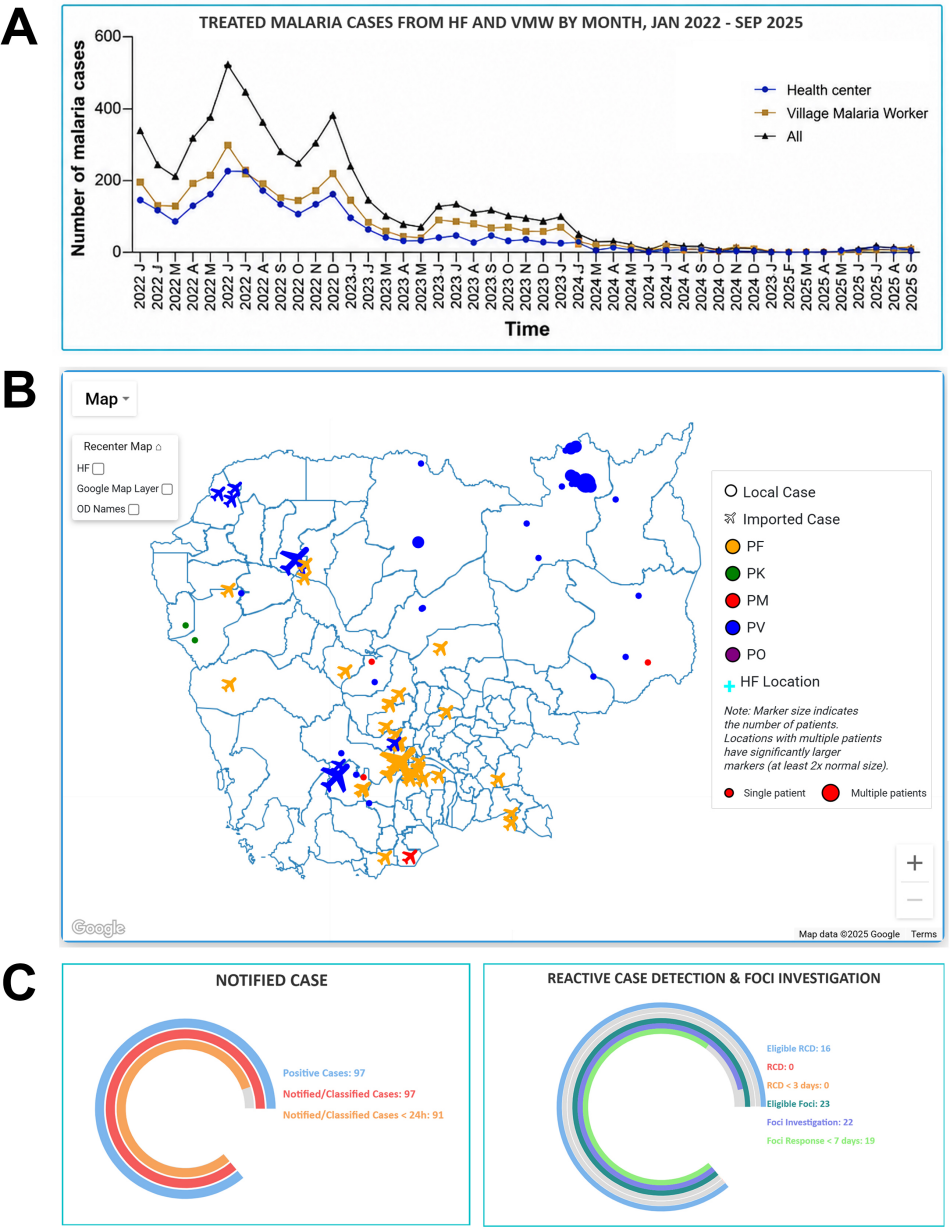
Snapshot of indicators of surveillance performance from the Malaria Information System dashboard. (**A**) Number of malaria cases and reporting route (January 2022 to September 2025). (**B**) Status of malaria cases in Cambodia (January to September 2025). (**C**) Performance against 1-3-7 targets (January to September 2025). These outputs are automatically generated from data submitted via the health center and VMW apps and included on the dashboard. The figure was derived with permission from CNM’s online malaria information system available via their website [36].

The MIS aggregates case data for all reported malaria cases in Cambodia. Malaria cases continued to decline in 2025 compared to previous years (Figure 5A), with no malaria deaths since 2018. Between January 1, 2025, and July 31, 2025, 69 cases were reported: 23 locally acquired, 7 domestically imported (from other districts), and 39 internationally imported (Figure 5B). Of these, 16 were reported via the health center app, 14 via the VMW app, and 39 internationally imported cases were added by MIS staff as these cases occurred in non-endemic areas.

Among the 69 cases, 95.7% (66/69) were notified and classified within 1 day. Reactive case detection was completed within 3 days for 21 of 21 eligible cases. Of 19 eligible foci, a foci response was initiated within 7 days for 16 (Figure 5C), reflecting strong adherence to the 1-3-7 framework.

National roll-out of iDES was completed in 2020. In 2024, 91.3% (173/186) of eligible cases were enrolled. Follow-up was completed to Day 28 for 99.4% (172/173) of cases and for *P. vivax* cases 92.9% (158/170) completed Day 90 follow-up. In 2024, dried blood spots were collected on Day 0 for 80.9% (140/173) of cases and on Day 28 for 77.5% (134/173) of cases permitting later analysis for molecular resistance markers. Declining case numbers reduce the sensitivity of clinical outcome data for detecting resistance, increasing the importance of molecular resistance markers [37].

Real-time foci identification classifies villages as active (≥1 indigenous case in the current year), residual (cases within the past 1‐3 years), or cleared. As of August 2025, there were 27 active and 70 residual foci. Foci are prioritized for intensified elimination efforts, including increased testing, behavior change interventions, bed-net distribution, and forest pack allocation (eg, hammock nets, repellents, and educational materials) [32].

**Figure 6.**
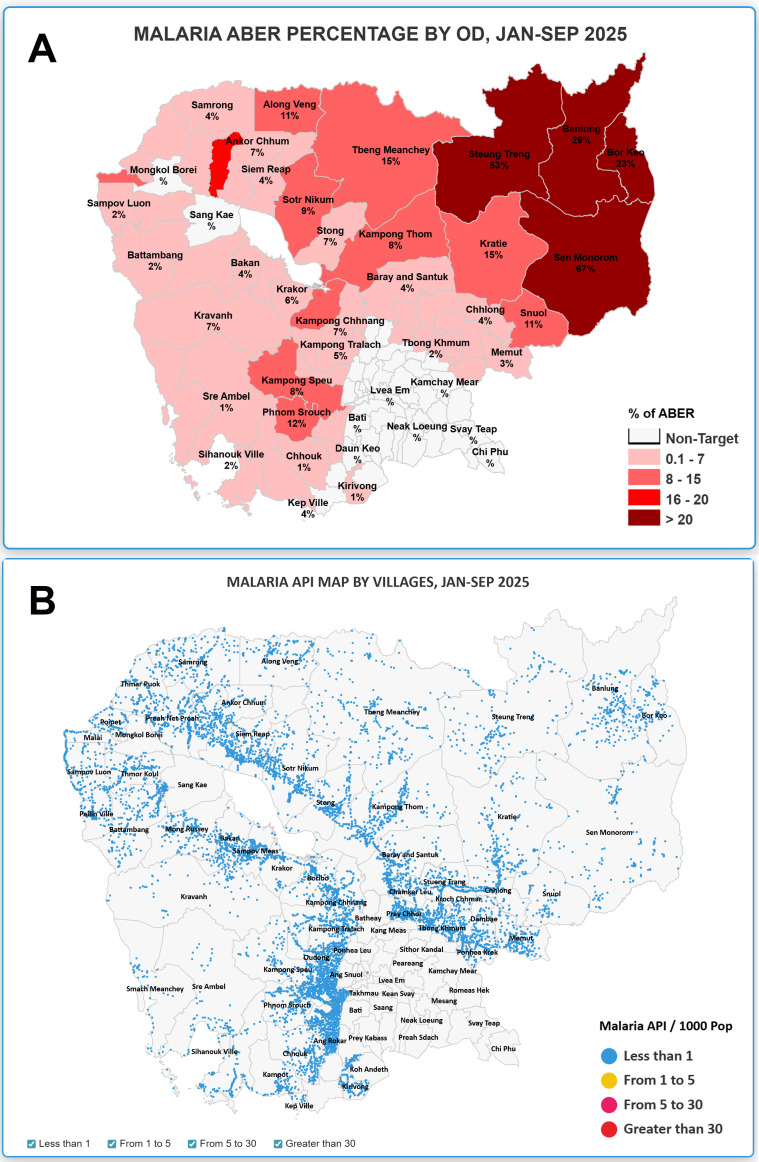
Core surveillance indicators for malaria stratification from the Malaria Information System dashboard. (**A**) Annual blood examination rate (ABER) (January to September 2025), defined as the number of blood samples tested, divided by the total population, then multiplying by 100. (**B**) Annual parasite index (API) (January to September 2025), defined as the number of confirmed malaria cases per 1000 people within a specific geographic area and time period. These outputs are automatically generated from data submitted via the health center and VMW apps and included on the dashboard. The figure was derived with permission from CNM’s online malaria information system available via their website [36].

Risk stratification ensures efficient resource allocation. In 2025, of 9.8 million residents in endemic areas, 22,119 were high risk, 147,602 medium risk, 2.8 million low risk, and 6.8 million at no risk. Stratification relies on the annual blood examination rate (ABER) and annual parasite index (API), both requiring comprehensive reporting and accurate population data. These metrics are automatically generated on the dashboard to the village level (Figure 6).

For ABER (Figure 6A), the proportion of the population tested annually indicates surveillance coverage. While ≥10% is recommended in control settings, elimination contexts require more targeted approaches, such as reactive testing and screening high-risk groups. Therefore, high ABER is expected in high-risk areas and lower in areas where risk is minimal. For API (Figure 6B), confirmed cases per 1000 people; measures transmission intensity and guides intervention focus. In elimination settings with low incidence, uneven surveillance can distort API, so village-level granularity is critical. Cambodia’s low API supports readiness for national elimination.

### User Feedback

**Figure 7.**
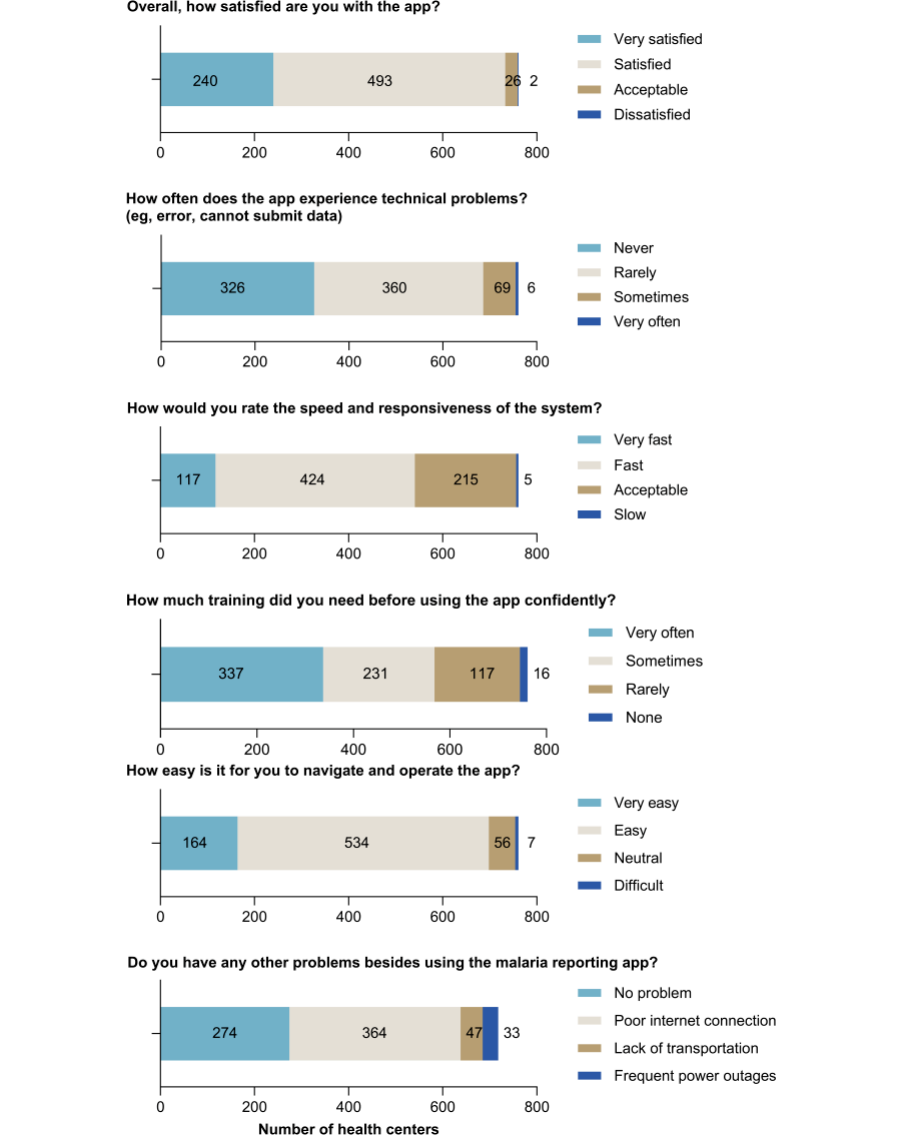
Health center user feedback on app technical performance and usability. The figure was derived with permission from CNM’s online malaria information system available via their website [36].

A usability and satisfaction survey conducted in July 2025 of all health centers received responses from 86.3% (761/882) of facilities within 1 month (Figure 7). Overall, 96.3% (733/761) of health centers were satisfied or very satisfied with the app, 90.1% (686/761) found that technical issues were rare or absent, and 71.1% (541/761) thought that system speed was fast or very fast. Although 44.3% (337/761) of centers confirmed that they needed regular training on the app, 91.7% (698/761) found navigating the app easy or very easy. Other common issues that impacted surveillance activities were poor internet connection, a lack of transportation to reach remote locations, and frequent power outages (Figure 7). This highlights the infrastructure constraints that had to be considered during the MIS system design.

The most recent in-depth survey was conducted in 9 randomly selected health centers across Battambang, Kampong Speu, and Mondul Kiri provinces (August-November 2024). Responses were obtained from the designated malaria focal person at each health center, all of whom had received standardized training on the use of the health center app and surveillance.

All 9 centers were aware of malaria reporting mandates, the requirement for case-based data submission, and national surveillance guidelines, demonstrating strong procedural awareness. All used both the health center app and CNM-issued paper forms (not processed, retained for audit purposes). All health centers confirmed immediate case reporting and compliance with WHO recommendations, including reporting zero cases, demonstrating high uptake and process adherence. Their understanding of surveillance objectives showed alignment with programmatic goals (Textbox 1) [20,21].

Textbox 1. **Summary of health center perceptions of the purposes of surveillance.**

- The system is designed to monitor malaria trends and track the number of tests conducted, regardless of whether they are few or many.
- It serves as a reminder and alert to keep health workers aware of the current malaria situation, helping them understand fluctuations over time.
- By tracking malaria cases, it enables identification of areas at risk and provides real-time data, making it easier to generate timely reports.
- Reports can be accurately managed at the health center level and efficiently shared with higher levels of the health system.
- The data are valuable for understanding where malaria cases are concentrated, whether in villages with many or few cases, and supports planning for the following month or year.

Self-reported comprehension of surveillance data was high: 100% at 3 centers, 80% at 6 centers. All found job-aids and SOPs clear, although one center noted complexity requiring further training. All centers knew where to seek support, indicating adequate system support. Eight of 9 centers conducted monthly data quality checks, all had supervision records; recent supervisory visits included data quality assessments, corrective actions, and written feedback, with standardized checklists used in 8/9 cases.

Staffing was adequate, with 6 centers reporting the correct number of staff (between 1 and 5 persons), 2 centers needed an additional staff member each, and 1 center had 1 more member of staff versus requirements. All centers had patient record books and tablets, with all tablets operational over the previous 30 days. This probably reflects the app’s device management module which supports a high level of functionality by quickly identifying technical issues. Three centers had computers and 5 had printers, with 1 computer and 2 printers fully functional for the last 30 days. Printers and computers are maintained locally, often with limited technical support. Infrastructure challenges persist: 4 centers experienced power issues, 3 had internet disruptions, and 2 had temporary system failures in the past 4 weeks. However, all centers reported access to troubleshooting support. Stock-outs pose a risk to elimination efforts and one Mondul Kiri center experienced a stock-out of RDTs and ACTs, highlighting the need for responsive surveillance systems in the preelimination phase.

**Figure 8.**
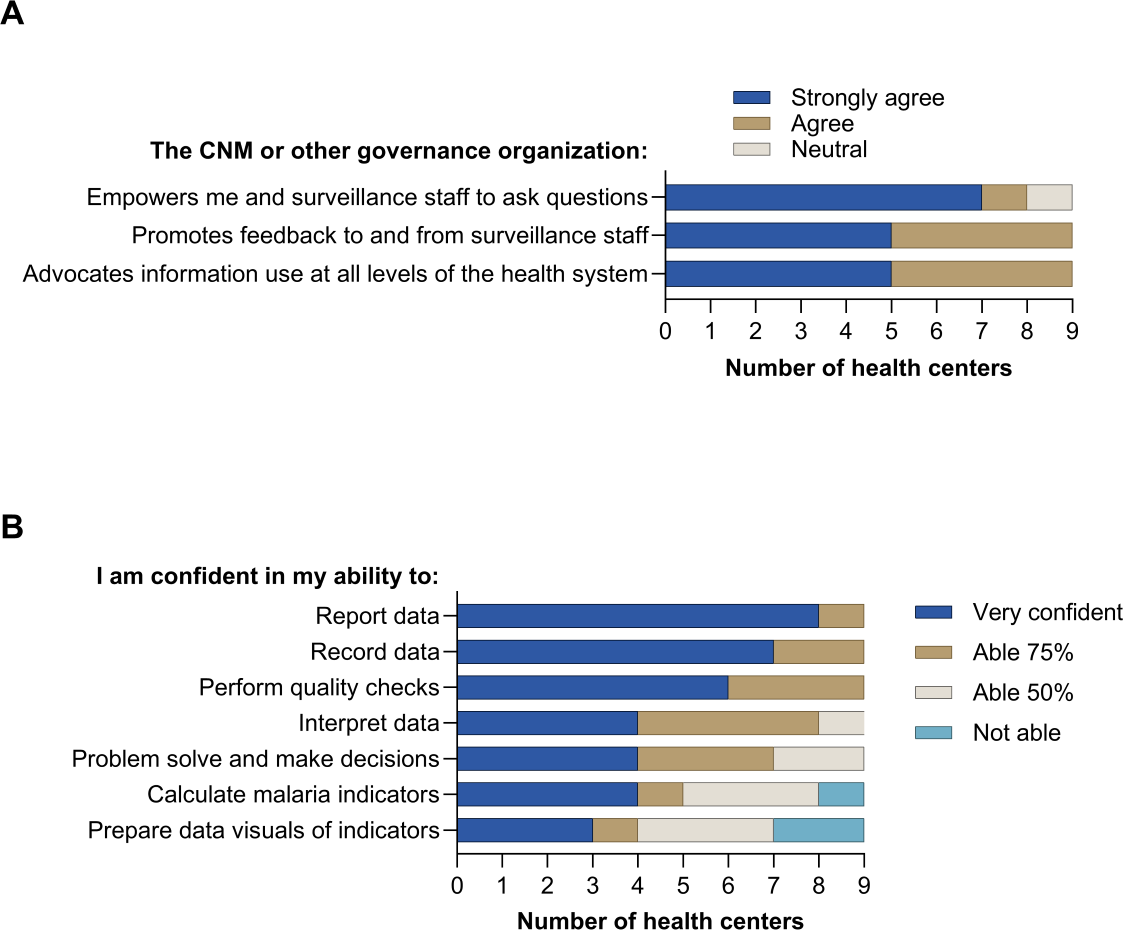
Health center user feedback on surveillance activities supported by the health center app. (**A**) Feedback on the supportive supervision of CNM (the National Center for Parasitology, Entomology and Malaria Control) regarding surveillance. (**B**) Confidence of users in their ability to conduct core surveillance activities, analyze data and interpret and use data. The figure was derived with permission from CNM’s online malaria information system available via their website [36].

All centers accessed dashboard visualizations and 4 downloaded data for further analysis. All held data review meetings, with bulletins produced in 6 centers. However, only 5/9 could calculate key indicators, with gaps in training and data availability reported in several centers. Despite this, 8 centers accessed data daily or weekly, and all at least monthly, and feedback on CNM and partner support was positive (Figure 8A). Consistent with the annual training cascade, all health centers received surveillance training in the previous 12 months on case notification, supervision, case data review, case report compilation, and recording of patient information. Six centers also received training on report preparation and 8 on data analysis and use. While confidence was high for data entry and quality checks, staff were less confident in calculating indicators and preparing visual outputs (Figure 8B).

## Discussion

### Study Findings and Generalizability

The Cambodian health center app constitutes a pivotal component of the national malaria surveillance system, enabling real-time, case-based data capture from endemic districts. By facilitating structured digital reporting at the point of care, the system improves the timeliness and completeness of malaria case notification, supports treatment adherence, and permits granular analysis of clinical and epidemiological data, enhancing case classification accuracy and enabling faster public health responses.

Effective surveillance prevents delayed treatment and onward transmission. The key strength of case-based data are alignment with the 1-3-7 surveillance framework [13,14]. This strategy contributed to malaria elimination in China, and similar approaches have been adopted in Cambodia, Thailand, Lao PDR, Myanmar, Viet Nam, India, Tanzania, and Zambia [13,14,38]. In Cambodia, digital surveillance at the point-of-care enables timely case management and follow-up, meeting 1-3-7 targets while integrating drug efficacy surveillance to ensure that treatments remain effective. At the same time, the Cambodian MIS fulfills WHO requirements for malaria elimination certification, ie, the surveillance system must be able to detect, investigate, and classify cases as locally acquired or imported [39]. Thus, no additional systems are required, avoiding duplication of effort in preparing for malaria certification.

While grounded in a specific country context, the challenges addressed by the health facility app are common across malaria-eliminating countries globally; malaria becomes increasingly spatially and temporally heterogeneous, focused in hard-to-reach communities. In Cambodia, geolocated case data have revealed persistent transmission in border and forested regions despite low overall case numbers, allowing tailored vector control and community engagement strategies [32,40]. Without sufficiently sensitive and sustainable surveillance, hard-won gains can be quickly lost following the withdrawal of control measures, as outbreaks in at-risk communities spread unchecked [41]. For example, in Sri Lanka in the 1960s, the Huai River basin in China in the 1960s and 1970s, and more recently in Thailand in 2017 [42-44]. Sub-national tailoring of interventions based on risk stratification informed by real-time surveillance allows targeting of residual foci and prompt identification and control of outbreaks, preventing resurgence [45].

As Cambodia moves toward a postelimination phase, emphasis will shift from local transmission detection to identifying imported cases, especially across porous international borders. Vigilance must be maintained and malaria should still be perceived as a threat both in terms of the risk of resurgence and for individual patients who may be at risk of poor outcomes if presentation, diagnosis and treatment are delayed [43,46]. The health facility app is integrated with complementary tools such as the VMW app, allowing detection in both formal health facilities and community settings. This digitally integrated approach allows the surveillance system to transition seamlessly from elimination to postelimination activities.

Sustainability is essential. Global funding is contracting, and as malaria cases decline, there is a risk of de-prioritization within national health budgets. The sustainable design principles applied to the Cambodian MIS and mobile reporting apps have broad applicability: national ownership, low recurrent costs, offline-first functionality, integration with routine workflows, efficient training requirements, and the ability to adapt rapidly to evolving epidemiological and programmatic needs while avoiding duplication. By contrast, a mobile case-based surveillance system in Myanmar struggled with long-term sustainability due to heavy reliance on external support, duplicated systems, and dependence on internet access [47-49].

In a broader context, the Cambodian MIS is an example of a successful digital health system implemented in a low-resource setting. Digital health tools show potential to widen care access and improve service quality, but in many low-resource countries progress has stalled at brief pilot stages with limited scale-up and unclear long-term viability [50]. A systematic review of digital health interventions in low-and middle-income countries identified barriers relating to infrastructure, equipment, internet access, electricity, and the nature of the intervention [51]. Facilitators were associated with stakeholder commitment and participation [51]. The experience of Cambodia illustrates that meaningful local involvement in design, deployment, and ongoing development is fundamental to context-specific problem solving and ensuring that systems remain flexible and robust in the face of evolving operational demands [52].

### Limitations

Despite the system’s strengths, challenges persist. While reporting and data entry have been widely adopted, gaps remain in health center staff’s capacity to analyze, interpret, and act on data. Although the dashboard offers summary indicators, users often lack the skills to conduct more complex analyses, even when data are exportable to Excel. Limited digital literacy and a lack of tailored analytic tools at the local level restrict the full use of surveillance data for decision-making.

Connectivity remains another constraint, particularly in remote areas. Although the system supports offline data entry, delayed synchronization still occurs, potentially affecting real-time response and 1-3-7 targets. Despite the expansion of internet access in Cambodia, this issue is clearly still impacting day-to-day surveillance, with most health centers reporting poor internet connectivity. Similar challenges are a common issue for digital health interventions in low-resource settings [51].

### Future Directions

Expanding the analytical capacity of the app to address evolving user needs represents a key area for development. As malaria transmission becomes increasingly isolated, analytics must become more specialized. Centralized generation of customized reports or automated analytics tailored to specific settings could enhance data use at health centers. Additionally, broadening the training curriculum to emphasize data interpretation and visualization skills would strengthen local decision-making.

Future system upgrades could include integration with other data sources, such as population mobility, environmental data, and socioeconomic indicators. Linking health and geospatial data with climate datasets may allow more accurate prediction of transmission risk and better outbreak preparedness [53].

Post-elimination, the ability to rapidly stratify malaria risk in terms of environmental receptivity, historical transmission, and importation rate is required for the prevention of reestablishment of malaria transmission. Incorporating machine learning and predictive analytics could enhance early warning capabilities, automate anomaly detection, and support more targeted public health interventions [54]. Given the existing integration of the MIS with the National Malaria Laboratory, there is also potential for further molecular surveillance to discriminate between local and imported infections [55].

Finally, expanding the platform to support surveillance for other diseases or syndromic conditions typically encountered at health centers may increase long-term sustainability, reduce duplication within the health system, and build broader public health resilience in Cambodia.

### Conclusions

Without urgent commitments from the GMS countries, resistant *P. falciparum* could have spread globally, leading to a resurgence in the disease. The considerable investment in surveillance and its contribution toward malaria elimination in the GMS requires greater examination and recognition. Also, the experience of Cambodia and other GMS countries provides valuable learnings and templates for designing flexible surveillance systems which are applicable in other malaria endemic settings transitioning from burden reduction to targeting malaria elimination. This is particularly relevant given the emergence of artemisinin partial resistance in Africa [1].

This report showed that strengthening the functionality and reach of Cambodia’s health center malaria surveillance app enhanced the efficiency and responsiveness of the national malaria elimination program. The app’s integration of case-based reporting, interactive dashboards, and training modules supports data timeliness, completeness, and use, transforming surveillance from a passive reporting task into an active, data-driven tool for decision-making.

The system’s national coverage and strong uptake among frontline health workers aim to capture every malaria case. Its open-source, in-house development model ensures adaptability to evolving operational needs, including new features for treatment adherence monitoring and foci response. Real-time data collection has facilitated high adherence to the 1-3-7 framework and informed targeted responses in residual transmission zones.

To ensure continued success and sustainability, future efforts should focus on strengthening analytic capacity among health center staff, addressing infrastructure limitations such as connectivity, developing predictive capabilities, and further integrating the app with other health and environmental information systems. Long-term sustainability will depend on continued political commitment, integration with surveillance for other health priorities, and active engagement of internal and external stakeholders. As Cambodia moves into the postelimination phase, a robust digital surveillance infrastructure is essential for protecting the population from malaria resurgence.

## Abbreviations

ABER: annual blood examination rate
ACT: artemisinin-based combination therapy
API: annual parasite index
CNM: National Center for Parasitology, Entomology, and Malaria Control
G6PD: glucose-6-phosphate dehydrogenase
iDES: integrated drug efficacy surveillance
MIS: Malaria Information System
RDT: rapid diagnostic test
VMW: village malaria worker
WHO: World Health Organization
